# Establishment of a Simple and Versatile Evaporation Compensation Model for *in vitro* Chronic Ethanol Treatment: Impact on Neuronal Viability

**DOI:** 10.3390/neuroglia3020004

**Published:** 2022-04-06

**Authors:** Meera Rath, Ariana M. Figueroa, Ping Zhang, Stanley M. Stevens, Bin Liu

**Affiliations:** 1 Department of Pharmacodynamics, University of Florida, Gainesville, FL 32610; 2 Department of Cell Biology, Microbiology and Molecular Biology, University of South Florida, Tampa, FL 33620

**Keywords:** ethanol, evaporation, compensation, cell culture model, chronic, neurons, viability

## Abstract

Alcohol overconsumption is a major cause of preventable mental disorders and death in the United States and around the world. The pathogenesis of alcohol dependence, abuse, and toxicity to the central nervous system remains incompletely understood. In vitro and cell culture-based models have been highly valuable in studying the molecular and cellular mechanisms underlying the contribution of individual CNS cell types to ethanol’s effects on the brain. However, conventional cell culture model systems carry the inherent disadvantage of rapid loss of ethanol due to evaporation following a bolus addition at the start of the treatment. We have established a multi-well cell culture plate-based ethanol evaporation compensation model that utilizes the inter-well space as a reservoir to compensate for the evaporative loss of ethanol in the cell treatment wells. Following a single bolus addition at the start of the treatment, ethanol concentration rapidly decreased over time. Through compensation using the multi-well plate platform, maintenance of ethanol concentrations ranging from 10–100 mM was achieved for up to 72 hours in a cell-free system. Greater effects in ethanol-induced decrease in neuronal cell viability were observed with than without compensation. Our method effectively compensates for the evaporative loss of ethanol typically observed in the traditional method. This method provides an economic, simple and effective in vitro model system for ethanol treatment over an extended timeframe where maintenance of a relatively constant concentration of ethanol is desired.

## 1. Introduction

Alcohol abuse is the third leading cause of preventable death in the United States [1] and alcohol use disorders are one of the most prevalent mental disorders globally that impact 8.6% of men and 1.7% of women [2]. In spite of significant effort over the decades, much remains to be gained in the understanding of the pathogenesis of alcohol abuse and neurotoxicity related to chronic alcohol use. Cell culture-based in vitro models that utilize immortalized cell lines or primary cultures derived or isolated from organs of interest are invaluable tools for mechanistic and experimental therapeutic studies of alcohol research, complementing well with far more complicated and costly studies performed in animals such as rodents [3, 4]. Compared to in vivo studies, cell culture-based in vitro models provide a number of unique advantages for mechanistic studies. First, the direct effects of ethanol on a cell type of interest can be distinguished from the secondary effects of mediators released from the neighboring cells. For the same token, the direct effects of a potential therapeutic agent in a cell type bearing the putative target can be evaluated to guide studies in more complex animal models. Second, through the utilization of co-culture systems and organotypic cultures, effect of ethanol on multiple and relevant cell types can be studied to reveal mechanistic pathways that may also be associated with alcohol use disorders. [5–7].

Cell culture-based in vitro ethanol treatments usually employ a bolus addition of a calculated amount of the chemical of interest at the start to achieve the desired concentration for treatment. At lower concentrations, one time and bolus addition of ethanol at the beginning of the treatment has been shown to alter a variety of cellular functions including intercellular redox state, membrane fluidity, lipid raft composition, transcellular and paracellular permeability [8–11]. At higher concentrations, ethanol-induced cytotoxic effects are more pronounced [12–17]. Additionally, brain glial cell functions have been shown to be affected at various ethanol concentrations [18–22].

For volatile, though water-soluble compounds such as ethanol, a considerable portion of the bulk of the initially added compound could be lost to evaporation, especially over an extended treatment time period to model a more chronic exposure scenario in vitro, leading to a lower than the intended amount available to the cells during treatment. Similarly, evaporative loss of ethanol during in vitro treatment might also have hindered the interrogation of effects of ethanol on cellular functions at concentrations close to the legal limit of 80 mg/dL, or 17.3 mM that are known to impair motor and/or executive functions in humans and animal studies [23].

In this study, we characterized the evaporation of ethanol in a multi-well cell culture format after a bolus addition at the beginning of the treatment. We also determined the amounts and timing of ethanol needed to be added to the non-treatment wells to compensate for the evaporative loss for a time period up to 72 hrs. Finally, we compared the effects on cell viability of treatment of immortalized human neuronal cells with ethanol with and without compensation.

## 2. Materials and Methods

### 2.1 Materials

Six-well cell culture plates (Costar 3516, Corning Life Sciences, Corning, NY, USA), Dulbecco’s modified eagle medium (DMEM, Corning 10–013-CV) containing 1 mM NaH_2_PO_4_ and 44 mM NaHCO_3_), heat-inactivated fetal bovine serum (FBS, Corning 35–011-CV), penicillin and streptomycin (Corning 30–002-CI), trypsin/EDTA solution (0.25%/2.21 mM, Corning 25–053-CI), ethanol (190 proof, Decon Labs 2801, King of Prussia, PA), MTT [3-(4,5-dimethylthiazol-2-yl)-2,5-diphenyltetrazolium bromide, ThermoFisher M6494) were obtained from ThermoFisher Scientific (Waltham, MA, USA). Alcohol dehydrogenase (Sigma A3263), nicotinamide adenine dinucleotide hydrate (NADH, Sigma N1511), glycine (Sigma 50046) were obtained from MilliporeSigma (Burlington, MA, USA). The human SH-SY5Y dopaminergic neuronal cells [24, 25] were obtained from American Type Culture Collection (Manassas, VA, USA).

### 2.2 Ethanol evaporation compensation model

**Figure 1.**
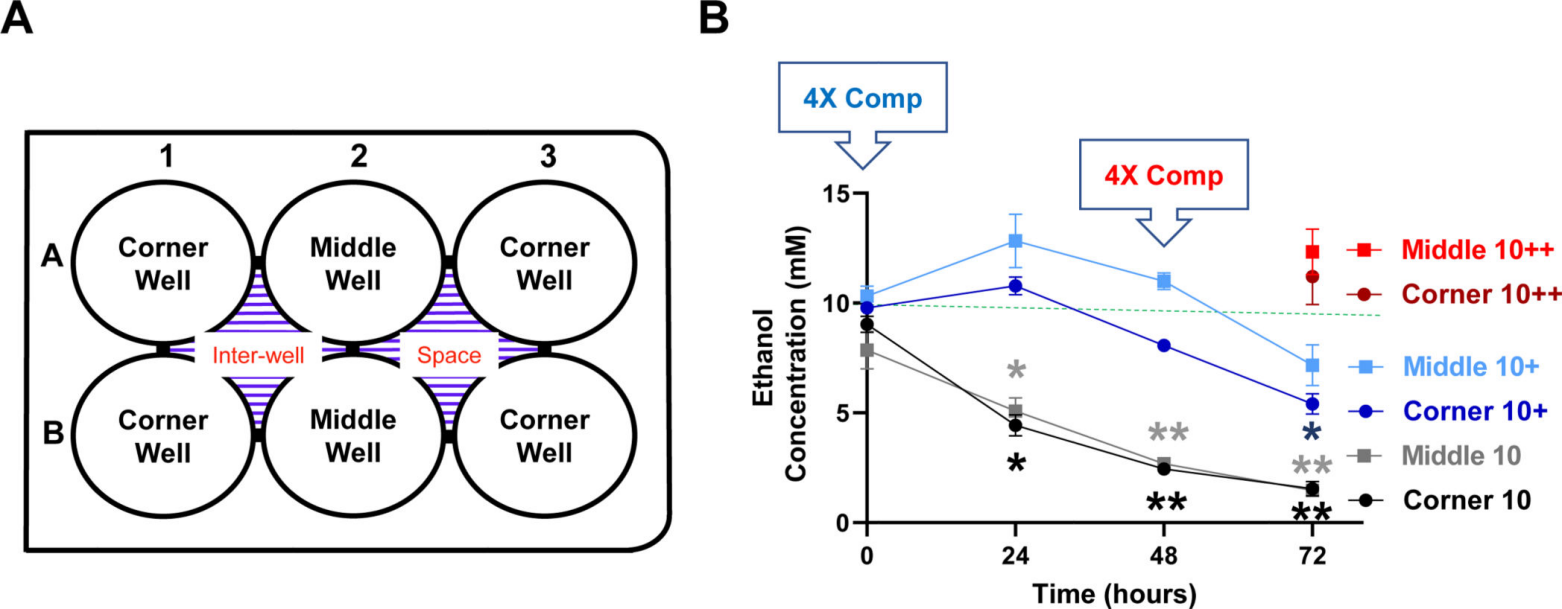
Time-dependent evaporation of ethanol with a starting calculated concentration of 10 mM and effects of compensation. **A.** At the start of the experiment (0 hour), ethanol (10 mM) was added to wells of 6-well culture plates (3 ml of culture media/well) and the plates were incubated in a CO_2_ cell culture incubator in a humidified environment with 5% CO_2_ and 95% air. **B.** At the indicated timed points, ethanol concentrations in the corner and middle wells were measured (black lines). In separate 6-well plates, additional ethanol was added to the inter-well space to 40 mM for evaporation compensation (4X Comp) at the start (blue lines, +) or at the start and again at the 48-hour time point (4X Comp, red symbols, ++) and ethanol concentrations were measured at the indicated timed points. The green dash line indicates the target ethanol concentration (10 mM). Results are expressed as mean ± SEM of the average of the two middle and four corner wells from 3 independent experiments. * and **, p < 0.05 and 0.005 respectively compared to the calculated 10 mM starting ethanol concentration.

To characterize evaporation during treatment and determine the amount and frequency of compensation needed, for each ethanol concentration (10, 25, 50, or 100 mM), a pair of 6-well cell culture plates (Figure 1A) were used. For any given calculated concentration of ethanol, a predetermined amount of ethanol was mixed with DMEM supplemented with 1% FBS, 50 units/ml penicillin and 50 μg/ml streptomycin. To each cell culture-treated well of both plates, 2.5 ml of the prepared ethanol containing media was added. For the plate without evaporation compensation (designated as uncompensated plate), 4 ml of DMEM-1% FBS was added to the inter-well space of the plate. For the plate with evaporation compensation (designated as compensated plate), 4 ml of DMEM-1% FBS containing predetermined amount of ethanol was added to the two inter-well spaces located between the corner and middle cell culture wells of the plate. The compensated and uncompensated plates were then placed inside Termo Forma Model 3110 water-jacketed CO2 incubator (ThermoFisher) and kept at 37°C in a humidified atmosphere containing 5 % CO2 and 95 % air. To determine the concentration of ethanol in the culture wells, 10 μl of the ethanol-containing media were immediately removed, in duplicate, from each well before (0 h) and 24, 48, 72 h after the plates were placed in the cell culture incubator and mixed with 100 μl of ice-cold 0.6 M glycine in 1.5 ml microcentrifuge tubes kept on ice for ethanol concentration determination. If the addition of ethanol-containing media to the inter-well space at the start of the incubation (0 h) was insufficient to maintain the desired concentration of ethanol in the cell culture wells throughout the treatment period (up to 72 h), the spent ethanol-containing media in the inter-well space was changed to freshly prepared ethanol-containing media at the 24 or 48 h time points. All procedures were carried out aseptically in a sterile cell culture hood (biosafety cabinet).

### 2.3 Determination of ethanol concentrations

Ethanol concentrations were determined with the alcohol dehydrogenase assay following our previously described protocol [26, 27]. Briefly, the samples collected at 0, 24, 48 and 72 h were assayed immediately following sample collection. Ethanol concentrations of the samples were calculated based on a series of ethanol standards prepared in the same media as that used for the evaporation/compensation studies. The detection limit was determined to be 0.78125 mM of ethanol in media.

### 2.4 Determination of the effect of ethanol on cell viability

SH-SY5Y cells were grown in DMEM supplemented with 10 % FBS, 50 units/ml penicillin and 50 μg/ml streptomycin at 37°C in an incubator with a humidified atmosphere containing 5 % CO_2_ and 95 % air. Cells were detached with the trypsin-EDTA solution and seeded into wells of 6-well culture plates at 3 X 10^5^ cells/well. Two days later, cells in each cell culture plate were treated with the desired concentration of ethanol in complete media (2.5 ml/well) with and without evaporation compensation.

Cell viability was determined with the MTT assay following our previously described protocol [25, 28]. At the end of treatment, 10 μl of culture supernatants was removed, in triplicate, from each well for ethanol concentration measurement. Afterwards, 250 μl of 5 mg/ml MTT was added to each well and the cells were incubated with MTT for 30–60 min. Culture supernatants were then removed and 1000 μl of dimethyl sulfoxide were added to each well to dissolve the formazan crystals. The plates were shaken on a shaker for 15 min at room temperature and 150 μl of the mixture was transferred, in triplicate, to 96-well plate. Absorbance was measured at 550 nm using a Synergy HT multi-well plate reader (Bio-Tek Instruments, Winooski, VT, USA).

### 2.5 Statistical Analysis

Ethanol concentration data with and without compensation were analyzed for statistical significance by two-way ANOVA followed by Sidak’s post hoc test using Prism 8.1 (GraphPad, San Diego, CA, USA). Cell viability data were analyzed by one-way ANOVA followed by Dunnett’s multiple comparison test. A p value of < 0.05, was considered statistically significant.

## 3. Results

### 3.1 Characterization of ethanol evaporation and effects of compensation

To determine the evaporation loss of ethanol during in vitro incubation, 2.5 ml of ethanol-containing culture media were added to each well of a 6-well culture plates to achieve a calculated concentration of 10 mM and the plate was then kept in a 37° C CO2 cell culture incubator. To each of the inter-well spaces, 4 ml of ethanol-free culture media were added (Fig. 1A). Aliquots of the ethanol-containing culture media in the 6 cell culture wells were removed at 0, 24, 48 and 72 h for ethanol concentration measurements. As shown in Figure 1B, compared to the calculated starting concentration of 10 mM, the measured concentrations were markedly lower at the 24-h time points and continued to decline at the 48 and 72-h time points. Due to the differential locations of the wells, especially in reference to the inter-well places (Fig. 1A), the concentrations of the middle and corner wells were presented separately. The measured concentrations of ethanol for the middle wells at the 0, 24, 48 and 72-hour time points were 78.5%, 50.8%, 26.8%, and 14.7% of and corner wells 90.4%, 44.3%, 24.5%, 15.4% of the calculated starting concentration of 10 mM, respectively. Ethanol concentrations of the middle wells were not statistically different from that of the corner wells at any of the time points (Fig. 1B).

To compensate for the evaporative loss of ethanol during incubation, 4 ml of culture media containing 40 mM of ethanol (i.e., 4X of 10 mM) were added to each of the inter-well spaces at the start (0 h), in addition to the addition of 2.5 ml of culture media containing 10 mM ethanol to each of the 6 culture wells of the plate. The measured concentrations of ethanol in the middle wells at the 0, 24, 48, and 72-h time points were 103.1%, 128.3%, 109.9%, and 71.7%, and corner wells 97.8%, 107.8%, 80.6%, and 54.0%, of calculated starting concentration of 10 mM, respectively (Fig. 1B, blue lines). In an attempt to compensate for the greater than 25% reduction in measured ethanol concentration of the corner wells at the 72-h time point, a second compensation was applied by replacing, at the 48-h time point, the spent ethanol-containing media in the inter-well spaces with 4 ml of fresh media containing 40 mM ethanol. With this, the measured ethanol concentrations of the middle and corner wells were 123.3%, and 112.0% of the calculated starting concentration of 10 mM (Fig. 1B, red lines).

**Figure 2.**
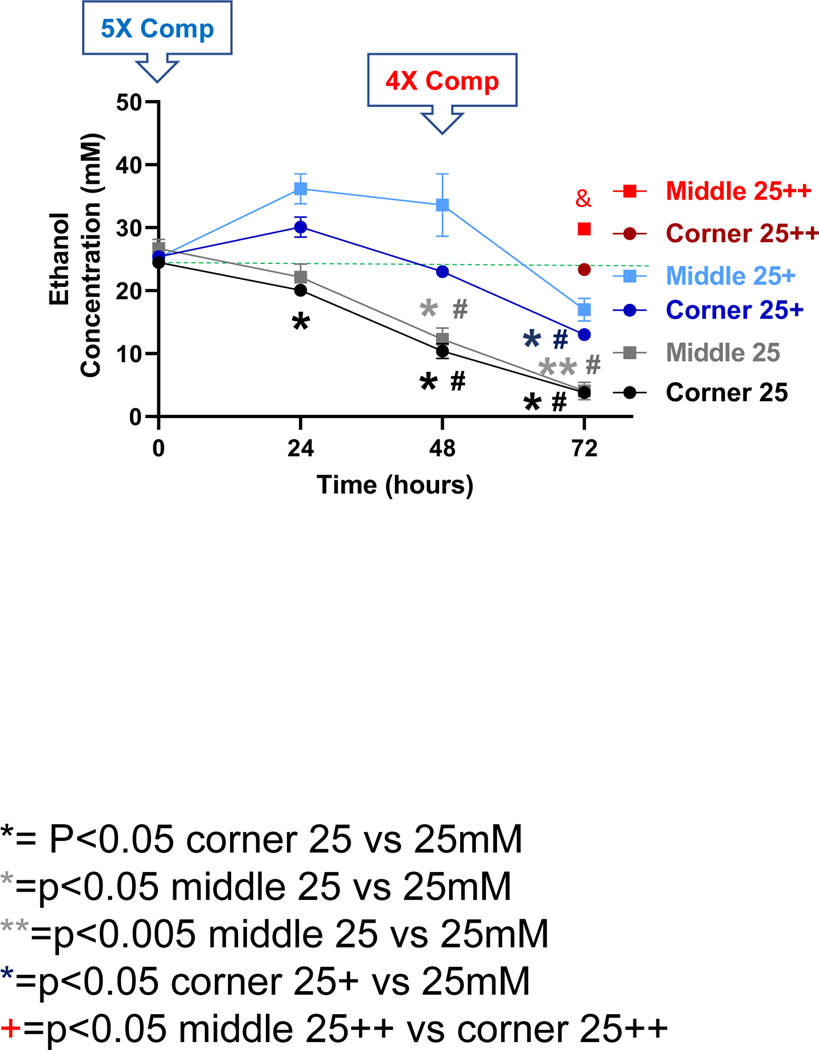
Ethanol evaporation and effects of compensation with a starting ethanol concentration of 25 mM. The magnitude of evaporation (black lines) was determined at the indicated timed points following a single bolus addition of a calculated concentration of 25 mM ethanol to the culture wells. The effects of compensation on the ethanol concentrations in the culture wells were evaluated by the addition of 125 mM ethanol at the start (5X Comp, blue lines, +) or 100 mM ethanol again at the 48-hour time point (4X Comp, red lines/symbols, ++) were evaluated. Results are expressed as mean ± SEM from 3 independent experiments. * and **, p < 0.05 and 0.005 compared to the calculated 25 mM starting ethanol concentration respectively; #, p < 0.05 compared to the preceding time point; &, p < 0.05 when comparing middle vs corner wells.

With a starting calculated concentration of ethanol at 25 mM, significant and time-dependent declines in the measured ethanol concentration were observed at the 24, 48 and 72-h time points for both the middle and the corner wells of the plate (Fig. 2, black lines). The measured concentrations of ethanol for the middle wells at the 0, 24, 48 and 72-h time points were 106.7%, 88.6%, 49.1%, and 16.2%, and the corner wells 97.9%, 80.3%, 41.6%, and 15.2% of the calculated starting concentration (25 mM), respectively. With a 1X (25 mM) compensation applied at the start (0 h), measured ethanol concentrations at the 0, 24, 48 and 72-h time points were 101.1%, 144.8%, 134.4%, and 67.9%, and of the calculated starting ethanol concentration (25 mM) for the middle wells, and 100.5%, 99.5%, 74.3%, and 65.9% for the corner wells, respectively (Fig. 2, blue lines). A second 4X (100 mM) compensation applied at the 48 h-time point resulted in the measured ethanol concentrations at the 72-h time point of 119.3% and 93.5% of the calculated starting ethanol concentration (25 mM) respectively (Fig. 2, red lines).

**Figure 3.**
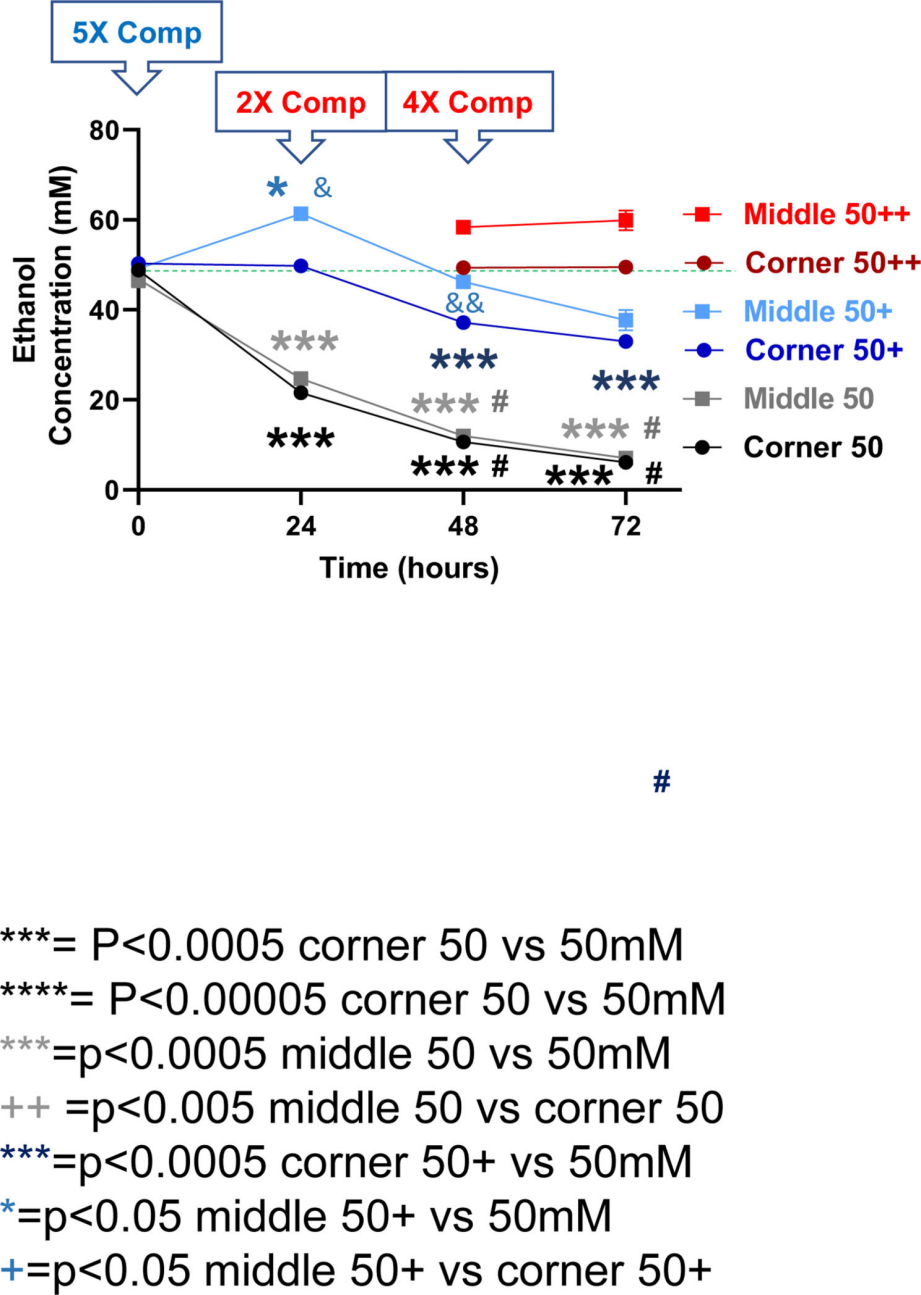
Ethanol evaporation and effects of compensation with a starting ethanol concentration of 50 mM. The magnitude of evaporation (black lines) was determined at the indicated timed points following a single bolus addition of a calculated concentration of 50 mM ethanol to the culture wells. The effects of compensation on the ethanol concentrations in the culture wells were evaluated by the addition of 250 mM ethanol at the start (5X Comp, blue lines, +) or 100 and 200 mM ethanol again at the 24 and 48-hour time points, respectively (2X and 4X Comp, red lines, ++) were evaluated. Results are expressed as mean ± SEM from 3 independent experiments. * and ***, p < 0.05 and 0.0005 compared to the calculated 25 mM starting ethanol concentration respectively; #, p < 0.05 compared to the preceding time point; & and &&, p < 0.05 and 0.005, respectively, when comparing middle vs corner wells.

Starting with a calculated concentration of ethanol at 50 mM, significant declines in ethanol concentration were observed at the 24, 48 and 72-h time points for both the middle and the corner wells of the plate (Fig. 3, black lines). The measured concentrations of ethanol for the middle wells at the 0, 24, 48 and 72-h time points were 93.7%, 49.4%, 23.9%, and 14.0%, and the corner wells 97.7%, 43.1%, 21.3%, and 12.2% of the calculated starting concentration (50 mM), respectively. With a 5X (250 mM) compensation applied at the start (0 h), measured ethanol concentrations at the 0, 24, 48 and 72-h time points were 98.4%, 122.8%, 92.4%, and 75.4%, and of the calculated starting ethanol concentration (50 mM) for the middle wells, and 100.5%, 99.5%, 74.3%, and 65.9% for the conner wells, respectively (Fig. 3, blue lines). A second 2X (100 mM) compensation applied at the 24-h time point resulted in the measured ethanol concentrations at the 48-h time point of 116.7% and 98.7% of the calculated starting ethanol concentration (50 mM) respectively (Fig. 3, red lines). A third and 4X (200 mM) compensation applied at the 48-h time point resulted in the measured ethanol concentrations at the 72-h time point of 119.8% and 98.9% of the calculated starting ethanol concentration (50 mM) respectively (Fig. 3, red lines).

**Figure 4.**
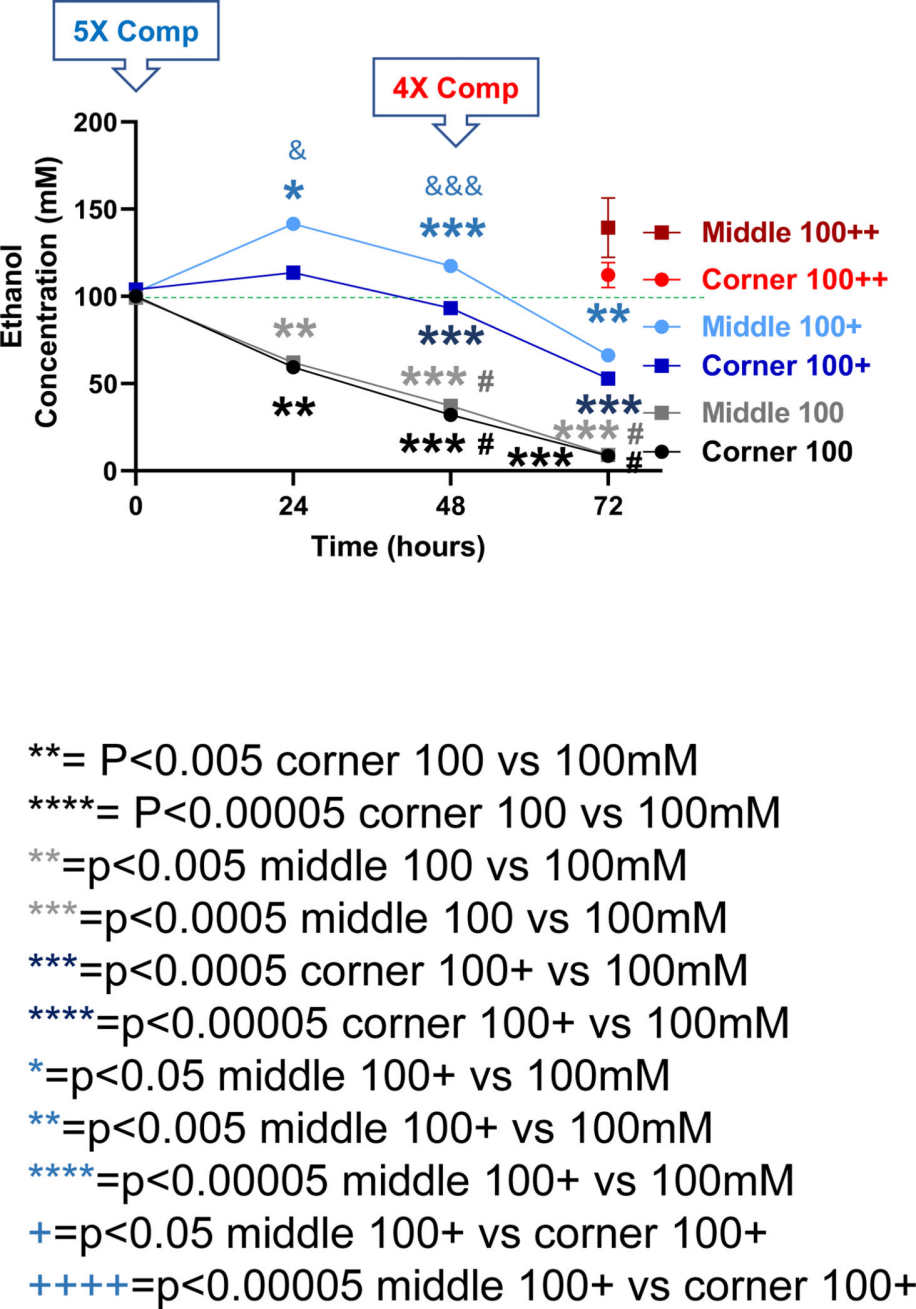
Ethanol evaporation and effects of compensation with a starting ethanol concentration of 100 mM. The magnitude of evaporation (black lines) was determined at the indicated timed points following a single bolus addition of a calculated concentration of 100 mM ethanol to the culture wells. The effects of compensation on the ethanol concentrations in the culture wells were evaluated by the addition of 500 mM ethanol at the start (5X Comp, blue lines, +) or 400 mM ethanol again at the 48-hour time point (4X Comp, red lines, ++) were evaluated. Results are expressed as mean ± SEM from 3 independent experiments. *, **, *** and ****, p < 0.05, 0.005, and 0.0005 respectively compared to the calculated 25 mM starting ethanol concentration; #, p < 0.05 compared to the preceding time point; & and &&&, p < 0.05 and 0.0005, respectively, when comparing middle vs corner wells.

For the starting calculated ethanol concentration of 100 mM, significant declines in ethanol concentration were observed at the 24, 48 and 72-h time points for both the middle and the corner wells of the plate (Fig. 4, black lines). The measured concentrations of ethanol for the middle wells at the 0, 24, 48 and 72-h time points were 99.3%, 62.1%, 37.2%, and 9.1%, and the corner wells 100.1%, 59.5%, 32.0%, and 8.5% of the calculated starting concentration (100 mM), respectively. With a 5X (500 mM) compensation applied at the start (0 h), measured ethanol concentrations at the 0, 24, 48 and 72-h time points were 102.4%, 141.5%, 117.4%, and 66.3%, and of the calculated starting ethanol concentration (100 mM) for the middle wells, and 103.9%, 113.5%, 93.3%, and 52.9% for the corner wells, respectively (Fig. 4, blue lines). A second and 4X (400 mM) compensation applied at the 48-h time point resulted in the measured ethanol concentrations at the 72-h time point of 139.4% and 112.3% of the calculated starting ethanol concentration (100 mM) respectively (Fig. 4, red lines).

In addition to characterize the evaporation of ethanol in the cell culture wells of the 6-well plates and the effects of using the inter-well space as a reservoir to compensate for the evaporative loss of ethanol in the culture wells within the same 6-well culture plates, 6-well culture plates containing only culture media (blank plates) were placed next to the ethanol-containing culture plates in the same incubator. No measurable amounts of ethanol were detected in any of the wells of the blank plates placed next to plates in the same incubator with starting and target concentrations of 10–100 mM with and with and without additional ethanol added to the inter-well spaces. In addition, there was no visible difference in the color of the culture media between the uncompensated and evaporation compensated plates indicative of a lack of effect of evaporation compensation on the pH of the culture media.

### 3.2 Compensation for evaporation enhances the effects of ethanol on cell viability

**Figure 5.**
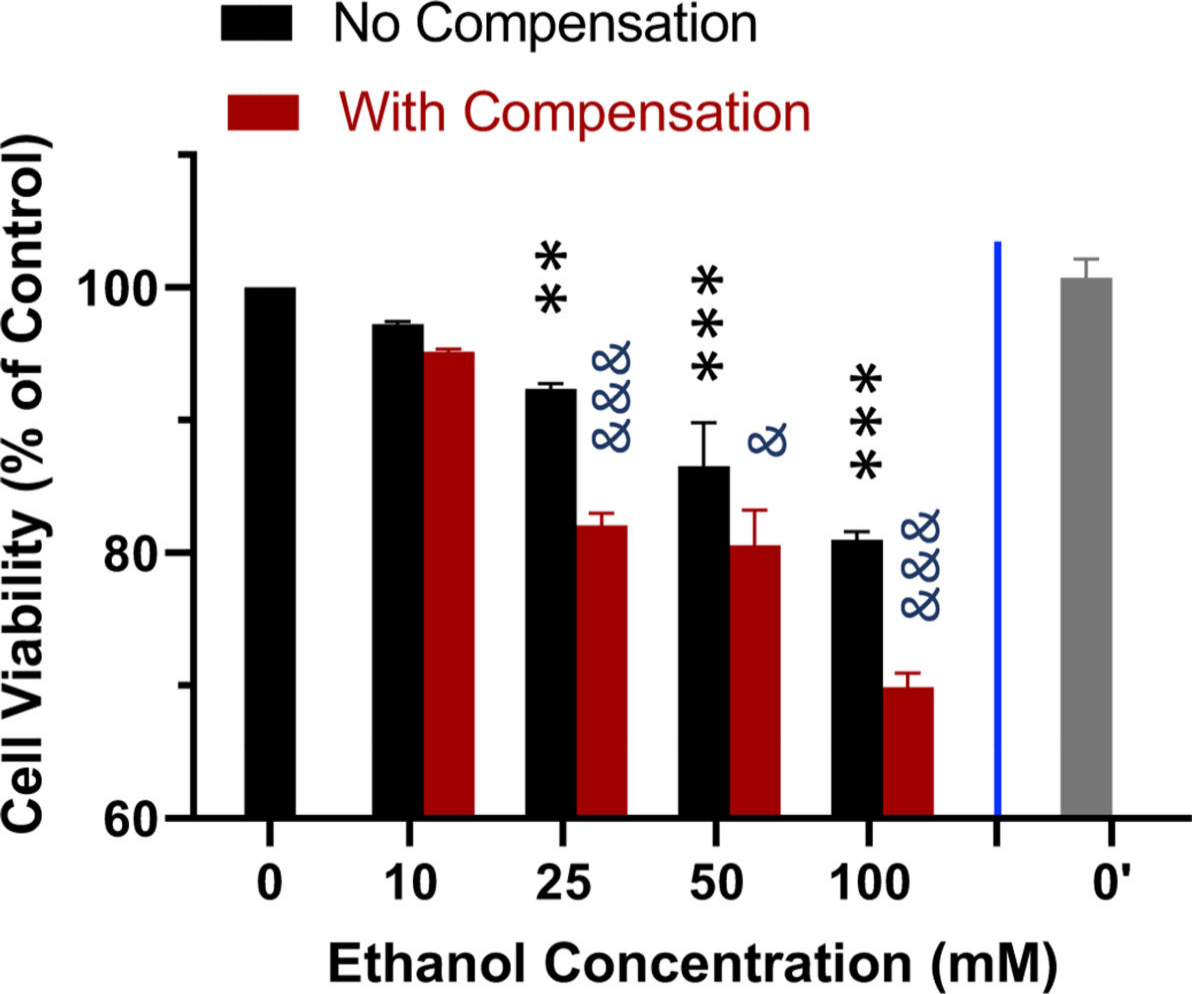
Effect of ethanol evaporation compensation on the viability of neuronal cells. Human SH-SY5Y dopaminergic neuronal cells were grown to near confluence in 6-well culture plates and treated for 24 hours with 10–100 mM ethanol with and without evaporation compensation. Cell viability was determined with the MTT assay and normalized against that of the media-treated control cells. Results are expressed as mean ± SEM from 5 independent experiments. ** and ****, p < 0.005 and 0.00005 respectively compared to the calculated media (0)-treated control cells; & and &&&, p < 0.05 and 0.0005 respectively no compensation vs with compensation. 0’ indicated the viability of the mock-treated control cells kept in a separate incubator.

The effect of ethanol evaporation compensation on cellular responses to ethanol was evaluated by comparing the effect of ethanol on the cell viability of the human SH-SY5Y dopaminergic neuronal cells. As shown in Fig. 5, treatment of cells for 24 h following a single bolus addition of calculated concentrations of ethanol ranging from 10 to 100 mM resulted in a concentration-dependent decrease in cell viability. Viability for cells treated with 10, 25, 50, and 100 mM ethanol decreased to 97.2%, 92.3%, 86.5%, and 80.9% of that of the control cells (Fig. 5, black bars). In contrast, when ethanol evaporation was compensated following the scheme described above (Fig. 3), viability for cells treated with 10, 25, 50, and 100 mM ethanol further decreased to 95.1%, 82.0%, 80.5%, and 69.9% of that of the control cells (Fig. 5, purple bars), with statistically significant enhancement of the effect of ethanol on cell viability at 25–100 mM. The viability of SH-SY5Y cells in additional control plates (i.e., without any ethanol treatments, 0’) that were kept in a separate cell culture incubator was not different from that of the control plates (0) kept in the same incubator as the ethanol-treated cells (Fig. 5).

## 4. Discussion

In this study, using a 6-well cell culture plate-based platform, we showed that the volatile nature of ethanol resulted in its rapid loss from the cell culture medium to evaporation during treatment, especially for extended timed periods (up to 72 hours) in an attempt to model the chronic effects of ethanol *in vitro* (Figs. 1–4). However, utilization of the inter-well space of the 6-well culture plate as a reservoir successfully compensated for the evaporative loss of ethanol (10–100 mM starting concentrations) in the cell-treatment wells such that a relatively consistent concentration of ethanol was maintained throughout the treatment period (Figs. 1–4). Moreover, a greater decrease in neuronal viability was observed when ethanol evaporation was prevented through compensation than when evaporation loss was allowed to occur (Fig. 5).

For this study, a range of ethanol concentrations from 10 to100 mM was selected with the lowest concentration of 10 mM [equivalent to 46 mg/deciliter (dL) or 0.046%] which is well below the equivalent legal limit of blood alcohol concentration (BAC) of 80 mg/dL of blood (i.e., 0.08% or 17.3 mM) in humans; the highest concentration of 100 mM (i.e., 460 mg/dL or 0.46%) was 5.7 times of the equivalent legal limit of BAC. This range of ethanol concentrations is relevant to ethanol concentrations that have been used in various in vitro studies usually with lower concentrations affecting cellular functions and higher concentrations impacting cell survival, especially that of vulnerable neurons [10, 13, 16, 20–22]. For example, treatment of hippocampal neurons with 10 mM ethanol, a level attainable following the consumption of a single alcoholic drink, has been shown to suppress neuronal long-term potentiation which is associated with memory and cognition [29]. Treatment of microglial cells with 50 mM ethanol induced non-proinflammatory protein expression profile changes associated with inhibition of phagocytosis [20]. Treatment of astrocytes with 75 mM ethanol reduced neurite outgrowth of co-cultured hippocampal neurons [30]. Treatment with up to 100 mM ethanol resulted in the preferential toxicity toward dopamine neurons [13]. The range of ethanol concentrations investigated in this study is also in line with equivalent blood alcohol concentrations (BAC) that have been detected in various animal models of alcohol administration [23, 26, 27, 31–34]. In voluntary binge drinking models resulting in post-consumption BACs averaging 60–70 mg/dl (~13–15 mM), locomotor impairment and anxiety-like behaviors were observed in mice [26] while increased motivation for ethanol self-administration was observed in rats [35]. In rats on a controlled binge drinking model, peak BACs as high as 230 mg/dl (~50 mM), decreased hippocampal neural progenitor cell populations were observed [32]. In a developing rat binge exposure model, no loss of hippocampal neurons was observed in rats with BAC of 39 mg/dl (~8 mM); yet significant neuronal loss was observed in rats with BACs of 190–360 mg/dl (~40–80 mM) [36]. Therefore, information provided by the current study on the evaporative loss of ethanol during *in vitro* treatment following a single bolus addition at the beginning of the treatment and the successful compensative measures to maintain a relatively constant concentration of ethanol throughout the treatment periods should be highly pertinent to investigators interested in study designs where in-treatment ethanol evaporative loss needs to be controlled.

In many instances, maintaining a steady level of ethanol during *in vitro* cell culture treatment to compensate for the evaporative loss has been highly desired and could, at the same time, be challenging [37]. Various measures to reduce ethanol evaporative loss from cell culture medium during in vitro treatment have been reported in the literature. For instance, by sealing the cell culture dishes with parafilm and supplementing the medium daily, ethanol loss during treatment was significantly reduced [37]. Rodriguez et al. reported a evaporation compensation model where cell culture dishes and an ethanol-containing dish were placed inside polystyrene boxes [38]. Borgs et al. compared the effectiveness of sealing culture plates with parafilm, tapes and a plate-sealing clamp system which was found to be a much more effective measure than parafilm or tapes in preventing evaporative loss of ethanol during treatment [39]. Kim et al reported the use of gel-filled capillary tubes as a source of ethanol for in vitro delivery in a 6-well culture format [40]. When in-culture ethanol concentrations were evaluated over a period of 144 hours, a bell-shaped curve was observed with a build-up time of approximately 36 hours at the front to reach a concentration between 150–200 mM that lasted for approximately 48 hours followed by a linear decline to near 0 mM by 144 hours [40].

The ethanol evaporation compensation model characterized and validated in this study is simple and versatile for several reasons. First, it is based on the widely-used multi-well cell culture plates and no special equipment and/or setup is required. It is also devoid of the use of sealing film or tapes that helps maintain the normal gas exchange and reduce the potential of microbial contamination. Second, the method is applicable to wide range of ethanol concentrations to be used for *in vitro* treatment of cells of interest. In the current study, the maintenance of 10–100 mM of ethanol at a relatively consistent level throughout the treatment periods of up to 72 hours was achieved. It is conceivable that the approach used in the current study can be readily adapted when concentrations of ethanol outside of this range is desired. Third, although the current method is based on 6-well culture plates, it does limit the growth of cells to be treated in the exact same format. For example, if the availability of cells is a limiting factor or if applications beyond cell harvest is desired, a much-reduced number of cells could be plated onto small-diameter cell culture coverslips or trans-wells that can be kept in the wells of the 6-well plates. Fourth, the utilization of the inter-well space within the confine of the same plate as the compensation reservoir makes it possible to house multiple plates of cells for treatments with different concentrations of ethanol in the same incubator. This would eliminate the need for an entire incubator to be dedicated to a single concentration of ethanol where an ethanol-containing vessel is placed inside the incubator as a compensatory reservoir. For starting and target concentrations of ethanol up to 100 mM characterized in this study, the presence of detectable amounts of ethanol was not detected in the media-containing culture wells of “blank” plates placed adjacent to the ethanol-containing plates. Furthermore, there was not a detectable difference in cell viability between the control cells (no ethanol treatment applied) in the plates kept in the same incubator as the ethanol-treated cells and the mock-treated control cells kept in a separate incubator (Fig. 5).

It should be pointed out that in this study, the time point for the measurement of ethanol concentration in the cell culture wells and the addition of ethanol to the inter-well space to serve as a reservoir for evaporative compensation was 24 hours apart. Ideally, a real-time and continual monitoring of the ethanol concentrations in the cell treatment wells would be most informative of the kinetics of the ethanol evaporation over time. By the same token, most desired would be a system that is capable of continuously delivering a precise amount of ethanol to the cell treatment wells, on demand, in response to the aforementioned continual ethanol concentration monitoring system. Also worth noting is that the middle culture wells, that is, those that were closer to the inter-well space, in general, had a higher ethanol concentration, especially in the case of evaporation compensation where the inter-well space was filled with culture media containing concentrations of ethanol several times higher than the starting/target ethanol concentration for cell treatment. This perhaps is not unexpected since the middle 2 wells were located in between the inter-well spaces on both sides. Nevertheless, even when the difference in ethanol concentration between the middle 2 and corner 4 wells was considered too large, one still has the access to 4 wells that have highly similar concentrations of ethanol.

The potential effect of the ethanol vapor from the inter-well space on the gas exchange over the neighboring cell culture wells in the current evaporation compensation model remains uncharacterized. Several studies reported in encased ethanol delivery systems such as tape-sealed culture plates and sealed plastic boxes, prolonged maintenance of cell cultures (e.g., >72 hours) has been shown to affect cell proliferation and viability [39, 41]. The effects were attributed to reduced/impaired gas exchanges although no quantitative measurements were reported in either study [39, 41]. In additional studies using similar enclosed systems, neither the impact on cell growth/viability nor gas exchanges of ethanol compensation were examined [37, 38]. This study utilized an open system based on the standard 6-well cell culture plate, which supported uninterrupted exchange of gases (CO_2_, O_2_) and vapors (humidified water and ethanol). Although no significant changes in media pH between the uncompensated and compensated plates were observed implying uncompromised gas exchanges, it would be ideal to document the local partial pressures for CO_2_, O_2_ in or above the wells of the multi-well plates during compensation to determine if the ethanol vapor from the compensation reservoir (i.e., the inter-well spaces) had any impact on gaseous exchanges. However, in-line and uninterrupted (in situ) measurements of the local gaseous partial pressure, as well as pH, in and above the wells of the culture plates would require highly sophisticated instrumentation which is currently unavailable to the investigation team.

The SH-SY5Y neuroblastoma cells are dopaminergic neuronal cells and have been widely used cell culture model to study dopaminergic neurodegeneration [25, 42]. Several studies have also reported reductions in the viability of SH-SY5Y cells following treatment with concentrations ethanol up to 500 mM [43, 44]. In this study, significant reduction in cell viability was observed following treatment with a bolus addition, at the start of treatment, of calculated amounts of ethanol for concentrations ranging from 25 – 100 mM (Fig. 5). A significantly greater reduction in cell viability was observed when ethanol evaporation was compensated compared to when it was not (Fig. 5). This observation thus validated the effectiveness of evaporation compensation during treatment of cells with ethanol across a range multiple concentrations with 25–100 mM tested in the current study. It is worth noting that a slight yet significant reduction in the viability of human dopaminergic neurons began to be observed at 25 mM (nearly 1.5 X of the legal BAC limit of 17.3 mM or 80 mg/dl) and a significantly greater reduction in cell viability was observed when evaporation compensation was put in place (Fig. 5). This observation is in line with the particular susceptibility of dopamine neurons to the ethanol-induced toxicity reported by others [13]. It also validates the notion that compensation for evaporation results in a greater quantity of ethanol available during treatment which leads to a greater degree of cellular toxicity. It should be pointed out that the concentration of ethanol (1.4 x of the legal limit of BAC) that caused a significant decrease in viability in a pure population of dopamine cells in vitro should not be interpreted as the concentration that would, for sure, cause significant neuronal loss in vivo. In fact, neuronal loss has often been observed following longer term and/or repeated exposure to ethanol that results in much higher BACs [32, 36] (also see above).

In conclusion, we have devised and characterized a simple and versatile ethanol evaporation compensation model that is capable of maintaining a relative constant level of ethanol during the entire treatment period. We validated the evaporation compensation enhanced effects of ethanol on cell viability. This model system can be readily adapted for *in vitro* studies of the effects of ethanol on various cells types.

## Data Availability

The data presented in this study are available on request from the corresponding author.
